# VoxelCoder: Classification of human cellular phenotypes via autoencoder batch alignment and hyperdimensional representation of cytometry data

**DOI:** 10.1016/j.patter.2026.101511

**Published:** 2026-03-26

**Authors:** Benjamin S. Mashford, Timothy Hewitt, Maryam May, Zixin Zhuang, Akshat Jain, Koula E.M. Diamand, Fei-Ju Li, Kristy Kwong, Stuart H. Read, Ainsley R. Davies, Dillon Hammill, T. Daniel Andrews

**Affiliations:** 1 The John Curtin School of Medical Research, The Australian National University, Canberra, ACT, Australia; 2 Computational Science Cluster, School of Computing, College of Systems and Society, The Australian National University, Canberra, ACT, Australia; 3 Australian Phenomics Facility, The Australian National University, Canberra, ACT, Australia

**Keywords:** cytometry, machine learning, bioinformatics

## Abstract

Technical variations in sample processing and instrument calibration, known as batch effects, can obscure true biological signals in cytometry data, impeding the integration of large-scale datasets. We present an autoencoder neural network approach that achieves batch correction comparable to that in existing methods while better preserving biological variation. Following alignment, cellular datasets are projected into a hyperdimensional voxel space that maintains interpretable marker-based features without requiring abstract latent dimensions, ensuring that identified cell populations remain fully interpretable. We benchmark this approach using a purpose-generated mouse splenocyte dataset with synthetic batch effects, demonstrating superior biological signal preservation compared to existing tools. Applied to clinical datasets, our method enables identification of cellular phenotypes associated with cytomegalovirus serostatus and COVID-19/sepsis discrimination, outperforming alternative approaches in downstream classification tasks. This framework addresses key technical limitations in integrating multi-batch cytometry datasets and provides a foundation for machine-learning applications in cytometry-based diagnostics.

## Highlights

• Autoencoder batch alignment removes technical variation, preserves biological signals
• Hyperdimensional voxel representation maintains interpretability
• Provides strong performance for disease classification on multi-batch clinical datasets
• Enables integration of cytometry data collected across different times and laboratories

## The bigger picture

Flow cytometry is a technology used in hospitals and research laboratories to analyze individual cells in blood and tissue samples. It helps doctors diagnose diseases, monitor immune responses, and guide treatment decisions. However, a persistent challenge has limited its potential for large-scale studies: when samples are processed at different times or in different laboratories, technical variations can mask true biological differences between patients. This makes it difficult to combine data from multi-center clinical trials or longitudinal studies where samples are collected over months or years. We introduce VoxelCoder, an artificial intelligence framework that removes these technical artifacts while preserving genuine biological signals. Our approach also represents cellular data in a way that remains fully interpretable to researchers and clinicians, avoiding the “black box” problem common to many machine-learning methods. By enabling reliable integration of cytometry data across different times and locations, this work could accelerate the discovery of cellular biomarkers for early disease detection, improve monitoring of patient responses to therapy, and support the development of personalized treatment strategies.

## Teaser

Technical variations between cytometry experiments can obscure true biological signals. Mashford et al. introduce VoxelCoder, a neural network approach that corrects these batch effects while maintaining interpretable cellular features, enabling more reliable disease classification from multi-batch clinical datasets.

## Introduction

Quantitative analysis of the cellular phenotype is routinely performed in a research and clinical setting through simultaneous measurement of cellular markers that target surface proteins, nucleic acids, and other molecular components. Common modalities of this routine cellular assay include both flow cytometry^1^^,^^2^ and cytometry by time-of-flight (CyTOF) mass cytometry.^3^ Two technical challenges limit the automated interpretation of these data: batch effects in data acquisition and the complexity of analyzing high-dimensional marker combinations.^4^ To counter these difficulties, current methodologies rely predominantly on manual gating, which, while established for standardized panels, becomes increasingly challenging as the number of measured parameters increases,^5^ as is now routinely possible with spectral flow cytometry.^6^

Batch effects in flow cytometry arise from multiple technical sources during data acquisition. These include variations in reagent preparation, such as differences in antibody clone selection and fluorophore conjugation between batches, as well as variations in reagent concentrations that affect staining intensity. Operator-dependent factors introduce additional variability through differences in sample preparation technique, instrument setup, and data-acquisition protocols. Furthermore, inherent drift in instrument calibration over time affects both laser intensity and detector sensitivity, leading to systematic shifts in fluorescence measurements. Despite implementation of standardized protocols and quality controls,^7^^,^^8^ these technical variations between experimental batches persist, manifesting as shifts in marker intensity distributions. These variations present particular challenges for data integration across multiple studies or institutions,^7^ especially in multi-center studies with the requirement to pool samples to improve overall study power. While computational approaches have demonstrated potential for addressing batch effects,^9^^,^^10^^,^^11^^,^^12^^,^^13^^,^^14^ much scope still exists for practical improvement of these methodologies.^4^^,^^5^^,^^15^^,^^16^^,^^17^

The increasing number of parameters measured by modern flow cytometers introduces additional analytical complexities.^6^ Standard manual gating in two dimensions requires examination of numerous scatterplots, potentially obscuring subtle correlations between markers.^7^ For instance, a six-marker panel necessitates analysis of 15 distinct two-dimensional plots, and this quickly increases with the addition of extra markers, becoming unfeasible for exhaustive visual analysis with the marker numbers now possible with spectral flow cytometry. Gating strategies to identify pre-defined cell populations of interest partially address this difficulty,^18^ yet this also works to obscure unexpected changes. Dimensionality reduction techniques have been applied to facilitate visualization and analysis of high-dimensional cytometry data.^9^ However, such approaches necessarily reduce project data into abstract latent spaces, potentially obscuring rare cell populations that may have biological significance.^16^

Recent computational methods have proposed alternative strategies for analyzing high-dimensional cytometry data. The adaptation of single-cell RNA sequencing analytical techniques to flow-cytometry data^19^ suggests new approaches to population identification. Additionally, investigations into hyperdimensional computing have demonstrated the feasibility of analyzing flow-cytometry data using interpretable marker-based features rather than abstract projections.^20^ Analysis of multi-parametric flow- and mass-cytometry datasets in hypervoxel space conceptualizes each individual cell as a data point in a multi-dimensional lattice. Each voxel, or hypercube, is a discrete region of the multi-dimensional parameter space, where each dimension is defined by variation of a single marker intensity. Hyperdimensional computing frameworks can efficiently encode and manipulate high-dimensional representations, facilitating the extraction of meaningful insights from noisy and heterogeneous data sources.^21^ One example of alignment of biological manifolds containing cytometry data employed a generative adversarial network,^22^ while further work in this area has used quadratic form cluster matching to allow multi-dimensional batch alignment.^23^

We present a computational toolset and an implementation that combines deep-learning-based batch alignment with systematic multi-dimensional cell-population analysis. This employs an autoencoder^19^ neural network architecture to perform batch correction against a reference distribution, effectively normalizing technical variations while preserving biological signals. Following batch alignment, the pipeline implements a “voxel-gating” strategy that systematically analyzes cellular populations across multiple marker combinations while maintaining interpretable marker-based features. This is achieved by dividing each marker’s expression range into discrete intervals and examining all possible marker combinations to generate comprehensive, unbiased representations of cellular phenotypes that remain fully interpretable. The resulting discretized population signatures can be used to build robust classification models for diagnostic applications. An implementation of this framework, named VoxelCoder, accompanies this work.^24^ We demonstrate that VoxelCoder outperforms traditional manual gating approaches while eliminating the need for per-plate normal controls and manual intervention. We evaluate VoxelCoder on a number of multi-color flow-cytometry and CyTOF mass-cytometry datasets and demonstrate classification performance while maintaining interpretability of results.

## Results

### Batch alignment

**Figure 1:**
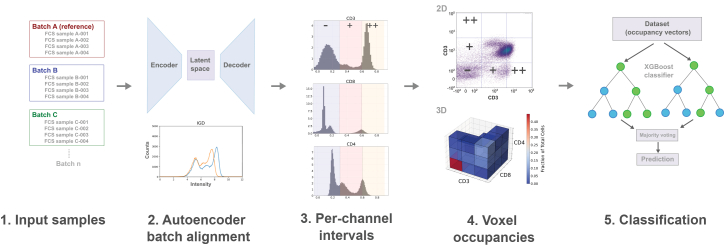
Overview of the VoxelCoder computational pipeline (1) Input cytometry samples from multiple batches. (2) Autoencoder-based batch alignment to a reference distribution. (3) Marker intensities discretized into fixed intervals (−, +, ++). (4) Voxel occupancy calculation across marker combinations. (5) Machine-learning classification for phenotype prediction.

We developed a new approach to integration of cytometry data that employs an autoencoder neural network architecture to perform batch correction (Figure 1). We employ this architecture using a randomly chosen reference batch of samples collected at a single time point to establish the target distribution. The autoencoder learns to transform input samples to match the reference batch distribution while preserving biological variation. The autoencoder is implemented with batch-normalization layers and is trained using both reconstruction loss and distribution matching via histogram loss. In brief, the network consisted of three encoding layers (64, 32, and 16 nodes) and three symmetrical decoding layers, with rectified linear unit (ReLU) activation functions between layers. The autoencoder was trained to transform input data from different batches to match a reference distribution. For a given multi-batch dataset, one batch is randomly selected as the reference, and a model is trained using a combination of mean squared error (MSE) loss and a custom histogram loss function that encouraged the transformed data to maintain marker distribution shapes similar to those of the reference batch. The histogram loss term is computed by comparing normalized histogram distributions between the network output and reference data across all markers, using Wasserstein distance as the similarity metric. This dual loss function helps ensure that both individual cell measurements and overall population distributions are preserved during batch alignment.

### Voxel gating

Following batch alignment, we introduced a systematic hypervoxel gating analysis strategy to comprehensively characterize cell populations across multiple markers. This method divides each marker’s expression range into three intervals (low, medium, and high; denoted −, +, and ++, respectively), then exhaustively analyzes all possible two- and three-marker combinations. For each combination, we compute cell occupancy frequencies across the resulting *n*-dimensional voxel space (e.g., the three intervals generates 3 × 3 × 3 = 27 voxels per marker combination in three dimensions), generating a high-dimensional feature vector that captures detailed information about cell-population distributions.

While conventional analysis of flow-cytometry data relies on manual gating strategies where cell populations are sequentially identified using two-dimensional projections of the data, such approaches are limited to examining pre-defined regions of interest and may not capture cell populations that exist in unexplored marker combinations. The hypervoxel gating methodology described here addresses these limitations while maintaining interpretability, like manual gating. The resulting hypervoxel occupancy features serve as input for subsequent machine-learning classification tasks, providing a comprehensive and unbiased representation of cellular phenotypes that may include both previously identified and potentially novel cell populations.

### Benchmarking of batch alignment

We evaluate our batch-alignment and voxel-gating approach with one purpose-generated benchmark dataset and two real-world datasets. The benchmark dataset is a biologically replicated, multi-color flow-cytometry experiment of C57BL/6 mouse spleen cells with a synthetic batch effect introduced between technical replicates by varying the dilutions of the antibodies (see methods). In addition to this, the real-world data are two public datasets, including (1) mass-cytometry data of latent cytomegalovirus (CMV) infection^25^ and (2) a multi-modal dataset of both flow and mass cytometry from individuals infected with COVID-19 and their clinical outcome.^26^^,^^27^ In evaluating the performance of batch alignment of these data, the hypervoxel gating strategy described above provides a ready framework for benchmarking.

### Alignment of synthetic batch effect

As mentioned, to appraise the performance of batch alignments, we generated a specific benchmark dataset with splenic cells from three wild-type C57BL/6 mice (see methods). These wild-type mice represent biological replicates, and for each of these a blood sample was analyzed in three technical replicates, where a synthetic batch effect was introduced by varying the concentration of antibodies included in the marker panel used for each of the three replicates (see methods, Figure 3, and Table 4). The panel included 11 markers: B220, CD3, CD8, CD19, CD25, CD44, CD62L, IgD, IgM, Ly6C, NK1, and a viability dye. Prior to analysis, data underwent standard pre-processing including compensation and debris removal. In this benchmark dataset, the technical replicates are derived from the same spleen cell drawn from the same individual mouse. A perfect batch alignment of the flow-cytometry information from the three replicates should result in a near-identical distribution of marker intensities and cell counts in hypervoxel.

**Figure 2:**
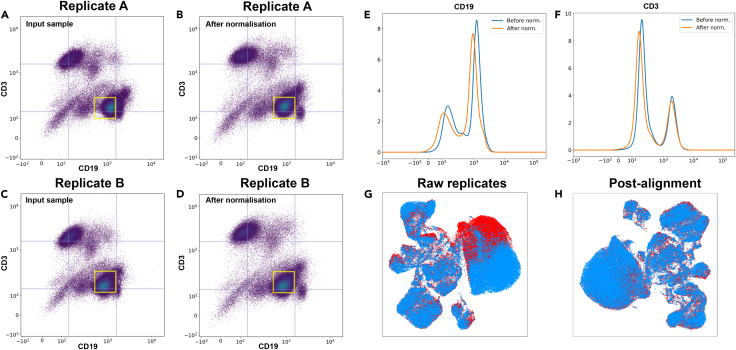
Batch alignment with an autoencoder neural network architecture (A–D) Two-dimensional scatterplots of CD3 and CD19 marker intensities for two technical replicates that harbor an inter-replicate synthetic batch effect introduced by varying antibody marker concentration. Replicate A (A and B) and replicate B (C and D) are aligned using an autoencoder model, as implemented in VoxelCoder. The squares in yellow are in a constant position between plots and shows the movement of the cells with batch alignment (between the input sample and the normalized output). The scatterplot color scale is proportional to cell density. (E and F) Histograms showing removal of batch-related shifts in signal intensities for two fluorescence channels for the CD19 (E) and CD3 (F) markers from the same replicate shown in (A) and (B). The blue lines indicate the distribution of intensities prior to batch alignment, and the red lines show the distribution of intensities after normalization. (G and H) UMAP visualization demonstrating batch alignment of two technical replicates (blue replicate superimposed over red replicate) with introduced synthetic batch effects. The raw replicate signals show clear batch effects (G), and post-alignment samples exhibit improved overlap of cell populations (H).

Figures 2A–2D show the effectiveness of our autoencoder-based batch-correction method across technical replicates of the same cell suspensions from three biological replicate mice. The technical replicates and the synthetically introduced batch effect they harbor is effectively removed by the autoencoder neural network described above. The input samples show clear batch-specific variations in both the CD3 and CD19 dimensions (Figures 2A–2D), particularly evident in the position and shape of the CD3^−^CD19^+^ B cell population (lower-right cluster with yellow box). The autoencoder successfully normalized this introduced batch variation while preserving the biological distinctions between cell populations. This preservation of the structure of cell populations is important, as it indicates that the method can distinguish between technical artifacts and true biological variation. The corresponding histogram analyses (Figures 2E and 2F) provide quantitative evidence of the normalization, showing how the method aligns both the location and shape of marker distributions to the reference batch. Notably, the correction handles non-linear distortions in signal distributions, as seen in the varying peak positions and shapes of the input histograms, suggesting that the method is robust to complex batch effects. The aligned outputs maintain consistent population boundaries across all three batches while preserving the relative proportions of major cell populations, demonstrating that the autoencoder learns a transformation that removes technical variation without compromising biological signal.

As shown in Figure 2G, prior to batch correction, cells from the same biological sample processed in different batches form non-overlapping clusters. After autoencoder-based alignment (Figure 2H), these cells merge, demonstrating successful normalization of batch effects while preserving the underlying cell-population structures.

### Comparison with existing toolsets

Additionally, we benchmarked this autoencoder neural network approach, implemented by VoxelCoder, against three popular batch-alignment methods: CytoNorm, CyCombine, and Harmony. Figure S1 shows a comparison of two-dimensional scatterplots before and after batch alignment. To benchmark these tools, we use two differing benchmarking approaches. The first is through comparison of the similarity of the distributions of intensities for each marker channel. For this, we used per-channel Wasserstein distances (Table 1). Complementary to this, we secondly employed hypervoxel-based Euclidean distances (Table 2). These complementary approaches provide a comprehensive assessment of batch-correction performance, with per-channel analysis focusing on individual marker distributions and hypervoxel analysis capturing high-dimensional phenotypic relationships.

**Table 1:** Wasserstein distances between technical replicate mice after batch alignment with different methods

Sample	Non-aligned	VoxelCoder	CytoNorm	CyCombine	Harmony
Mouse 1 (male)	0.080	0.081	0.085	0.060	0.055
Mouse 2 (female)	0.085	0.030	0.089	0.061	0.027
Mouse 3 (female)	0.052	0.024	0.061	0.036	0.028

**Table 2:** Euclidean distances calculated between technical replicate mice, using hypervoxel representation, after batch alignment

Sample	Non-aligned	VoxelCoder	CytoNorm	CyCombine	Harmony
Mouse 1 (male)	0.145	0.028	0.188	0.054	0.030
Mouse 2 (female)	0.180	0.043	0.288	0.060	0.032
Mouse 3 (female)	0.185	0.053	0.220	0.087	0.042

The removal of batch effects has two important components to consider. First, the distributions of signal values need to be brought to a similar mean and variance between batches to allow simplified direct comparison. Second, however, this must be achieved while also preserving the biologically relevant features that are the real signal differences between samples. For example, it is simple enough to fit any distribution present in an input dataset to a given mean and variance, yet this will likely erase the real signal present in the data. We compared the popular batch-alignment methods with our autoencoder normalization approach for both similarity of input distributions and preservation of biological signal following normalization.

The similarity of normalized distributions from different popular tools using per-channel distributions reveals that Harmony generally achieves the lowest Wasserstein distances across most mouse samples (Table 1). Wasserstein distance is a metric that represents the subtraction of one distribution from another and is colloquially known also as earth mover’s distance. Lower Wasserstein distance indicates effective alignment between technical replicates. VoxelCoder demonstrates competitive performance, particularly for certain samples, though it exhibits slightly higher distances for others. CyCombine also performs adequately, with consistent distances across all samples examined.

The hypervoxel analysis provides an assessment of batch-effect correction methods by characterizing cellular phenotypes in high-dimensional space, offering a complementary perspective to the per-channel Wasserstein approach. Using hypervoxel cell counts, we calculated the Euclidean distance between input and normalized counts using this multi-dimensional representation. Table 2 shows that both VoxelCoder and Harmony demonstrate effective performance across the samples tested, with Harmony achieving the lowest Euclidean distances for most samples, while VoxelCoder performs comparably well.

**Figure 3:**
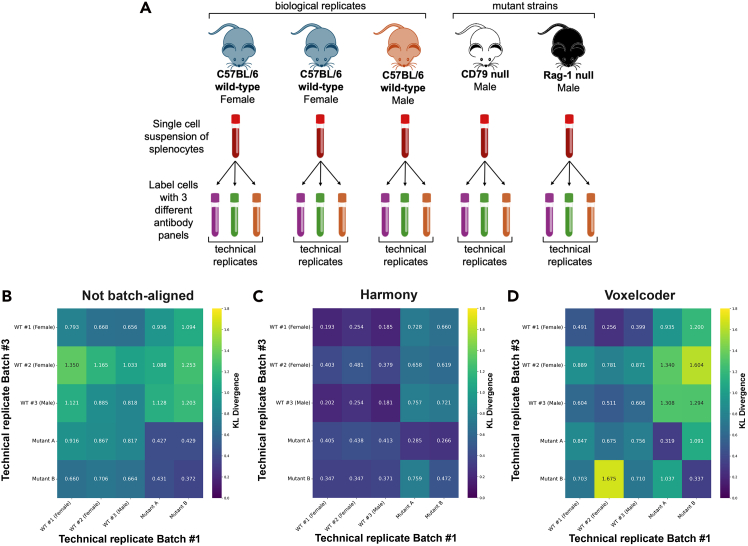
Experimental design and evaluation of biological signal preservation across batch-alignment methods (A) Schematic of design of synthetic batch-effect mouse dataset. Spleens from three individual wild-type C57BL/6 mice (one male, two female) provided cell samples for biological replication. A further two mutant C57BL/6 strains provided examples of biological variation due to genetic changes. Each suspension of splenocytes from each mouse was split into three technical replicate vials of equivalent total cell count. These technical replicates for each mouse were prepared for flow cytometry with one of three marker cocktails. The marker cocktails contained identical antibodies with deliberate, minor differences to antibody dilutions (Table 4) to introduce a synthetic batch effect from the same input cells per biological replicate. (B–D) Heatmap plots showing KL-divergence distances between five mice in the synthetic batch-effect dataset. Dataset includes three wild-type mice (WT #1, #2, #3) and two mice with significant mutant phenotype (mutant A and mutant B). Scores are derived from per-channel histograms using 50 bins per channel. The figure shows distances in non-batch-aligned dataset (B), compared to distances in Harmony-aligned (C) and VoxelCoder-aligned (D) datasets. Lower diagonal values indicate better technical replicate convergence; preserved off-diagonal values indicate retention of biological differences.

When considering retention of biological signal following batch normalization, we compared the interbiological replicate differences of the wild-type and mutant mice synthetic batch-effect data (Figure 3). These synthetic benchmarks provide a unique validation of the technical performance of the autoencoder framework. To quantify biological signal retention, we calculated Kullback-Leibler (KL) divergence between all sample pairs, where larger divergences between biologically distinct samples (wild-type vs. mutant strains) relative to technical replicates indicate better preservation of biological variation. The synthetic dataset design allows clear separation of biological from technical variation, since expected biological differences are known a priori, creating an ideal test bed for evaluating batch-correction methods.

While Harmony demonstrated aggressive batch mixing, our detailed analysis suggests that this comes at the cost of compressing biological variation (overcorrection). To quantify this trade-off, we calculated the ratio of mean diagonal to mean off-diagonal KL divergence values. In this framework, diagonal elements represent technical replicates that should converge after successful batch correction (lower values indicate better technical alignment), while off-diagonal elements represent biologically distinct samples that should remain separable (higher values indicate preserved biological signal). A lower ratio therefore indicates effective batch correction with preserved biological distinction. These findings were corroborated using kernel maximum mean discrepancy and energy distance metrics (Table S2). Harmony compressed the biological separation between distinct samples by approximately 49%, resulting in a diagonal/off-diagonal ratio of 0.70. In contrast, VoxelCoder maintained biological distinctness while correcting batch effects, achieving a superior ratio of 0.54. This preservation of global data topology was further confirmed by a higher Spearman correlation of the distance matrices for VoxelCoder (*ρ* = 0.77) compared to Harmony (*ρ* = 0.62). The risk of overcorrection was most evident in the distinct “Male Rag” outlier strain; while VoxelCoder improved the alignment of these replicates (KL reduction from 0.37 to 0.34), Harmony increased the divergence (from 0.37 to 0.47), suggesting that the algorithm forced the alignment of these biologically distinct cells onto unrelated wild-type populations. Together, these results suggest that VoxelCoder achieves batch alignment comparable to that of Harmony while better preserving biological signal, particularly in outlier populations where no true reference equivalent exists.

Computational requirements differed substantially between batch-correction methods. For the CMV dataset comprising 472 samples, VoxelCoder completed batch alignment in approximately 20 min, while Harmony required 70 min on the same hardware (Intel core i7-13700K processor with 16 cores and 24 threads, 64 GB RAM). VoxelCoder leveraged GPU acceleration (NVIDIA RTX 3060, 8 GB) for autoencoder training and transformation, contributing to the quicker processing time. Following batch alignment, conversion of the dataset to voxel representation required approximately 9 min on the same hardware.

### Identification of latent CMV infection through mass-cytometry data analysis

To assess our method against earlier work, using real-world data, we utilized a comprehensive CyTOF mass-spectrometry dataset.^25^ This dataset comprises 472 samples from nine independent studies, containing peripheral blood mononuclear cell measurements from healthy individuals along with their CMV serostatus. The dataset represents a challenging real-world scenario due to its heterogeneous nature combining data from multiple independent studies, making it particularly suitable for evaluating batch-alignment analytical methods for cytometry data.

**Figure 4:**
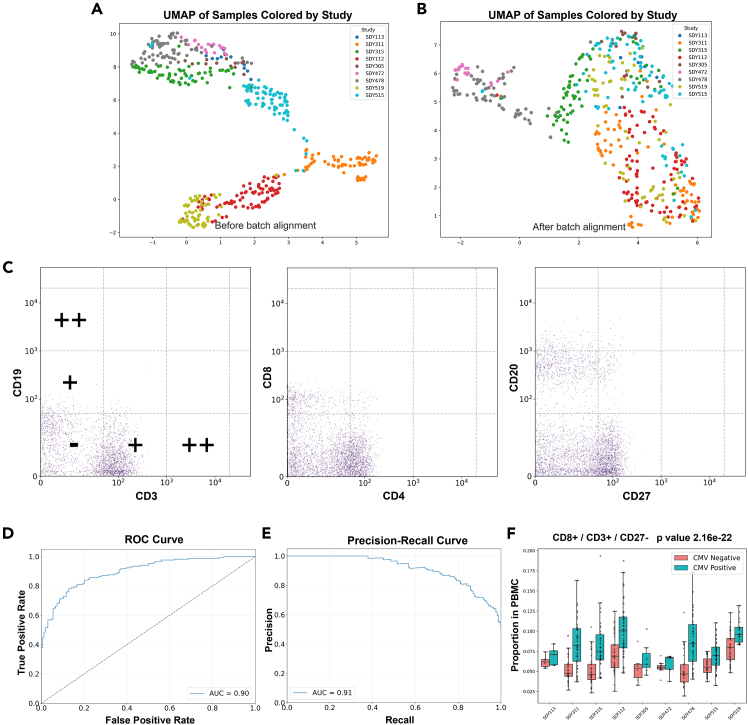
Batch alignment and classification analysis of cytometry data for CMV status prediction (A) UMAP visualization of samples colored by study batch before batch alignment, showing clear batch-specific clustering. (B) UMAP visualization after batch alignment, demonstrating improved mixing of samples across batches. (C) Representative flow-cytometry scatterplots showing population distributions and voxel-gating intervals for key markers (CD3, CD19, CD4, CD8, CD27, and CD20). (D) Receiver-operating characteristic (ROC) curve for CMV status classification showing an area under the curve (AUC) of 0.90. (E) Precision-recall curve for the classification model with AUC of 0.91. (F) Boxplots showing the proportion of CD8^+^/CD3^+^/CD27^−^ cells across different study batches, stratified by CMV status. Boxplots show median and interquartile range (IQR); whiskers extend to 1.5× IQR. Statistical significance was assessed by Mann-Whitney U test (*p* = 2.2 × 10^−22^).

We conducted a reanalysis of these data in a hypervoxel framework with VoxelCoder (Figure 4). Initial uniform manifold approximation and projection (UMAP) visualization (Figure 4A) clearly shows batch-specific clustering before alignment, with samples from each study forming distinct clusters. Following batch alignment with VoxelCoder (Figure 4B), we observe substantially improved mixing of samples across batches. Representative mass-cytometry scatterplots (Figure 4C) demonstrate our voxel-gating approach, showing how intensity intervals for key markers (CD3, CD19, CD4, CD8, CD27, and CD20) are partitioned into low (−), medium (+), and high (++) expression categories. These classifications form the basis for the hypervoxel framework used in our subsequent analysis. The receiver-operating characteristic (ROC) curve (Figure 4D) shows that our VoxelCoder-based model achieves an area under the curve (AUC) of 0.90 for CMV status prediction, outperforming the Harmony-aligned voxel analysis, which achieved an AUC of 0.82 (Figure S3). We further validated robustness through sensitivity analysis, demonstrating that classification performance remained high (AUC ≥ 0.80) regardless of reference batch selection (Figure S4).

Our reanalysis provides deeper phenotypic resolution than the original work and identified several highly significant CD8^+^ T cell populations that share key features with the original findings. The most significant hypervoxel combination (Mann-Whitney U test, *p* < 10^−20^) identified a cell subset with a CD8^+^/CD3^+^/CD27^−^ phenotype, which is concordant with the original work, as shown in Table 3 and the boxplots in Figure 4F. These plots clearly demonstrate the increased proportion of this specific T cell phenotype across different study batches in CMV-positive individuals compared to CMV-negative individuals. Notably, while Hu et al.^24^ emphasized CD94 expression, our analysis highlights CD27 downregulation combined with CCR7 downregulation as the dominant predictive features. The top five predictive marker combinations all featured CD8^+^ cells with low CD27 expression. The convergence of these features demonstrates that a simplified phenotypic signature centered on CD8^+^/CD27^−^/CCR7^−^ expression is sufficient to robustly classify CMV serostatus across heterogeneous cohorts.

**Table 3:** Top five most statistically significant voxels by CMV status discrimination

Feature	*p* value	Minimum occupancy	Maximum occupancy
CD8^+^/CD27^−^/CD16^−^	1.37e−22	0.004	0.289
CD8^+^/CD3^+^/CD27^−^	2.16e−22	0.003	0.290
CD8^+^/CD27^−^/CD38^−^	2.45e−22	0.004	0.265
CD8^+^/HLADR^−^/CD27^−^	3.46e−22	0.007	0.213
CD8^+^/CD27^−^/CCR7^−^	1.56e−21	0.008	0.300

Statistical significance assessed by Mann-Whitney U test comparing CMV-positive and CMV-negative groups. Calculated with VoxelCoder-aligned dataset.

### Discrimination between sepsis and COVID-19 patients using CyTOF and multi-color flow cytometry

As a further test with real-world data, we partially reanalyzed the cytometry dataset generated by the COMBAT consortium^26^^,^^27^ from individuals infected with COVID-19. This dataset presents significant analytical challenges typical of clinical cytometry data: high patient-to-patient variability, complex disease states, and technical variation across multiple collection sites. We reanalyzed their cytometry dataset using our VoxelCoder pipeline, demonstrating that automated multi-dimensional analysis can extract additional immunological insights and achieve robust disease classification.

The flow-cytometry measurements were made across six experimental batches from individuals clinically classified with mild, severe, and critical COVID-19 infection. The dataset also contains flow samples from patients with sepsis and flu, as well as healthy control samples (Table S1). To simplify the analysis task, we only focus on the following sample classes: (1) healthy, (2) critical COVID infection, and (3) sepsis. Before sample classification and biomarker discovery analysis were made, we applied our autoencoder batch-alignment model to the entire dataset.

In a hypervoxel framework using VoxelCoder, we developed a classification model to predict the three classes of infection status. The classification model achieved strong performance in distinguishing between patient groups, with area under the ROC curve values of 0.99, 0.90, and 0.92 for healthy controls, COVID-critical, and sepsis patients, respectively (Figure S2). Analysis of classification accuracy revealed perfect discrimination of healthy controls (100% correct classification), while COVID-critical and sepsis cases showed some degree of overlap, with 78.6% correct classification of COVID-critical cases and 83.3% correct classification of sepsis cases. This overlap may reflect shared immunological features between these acute inflammatory conditions.

**Figure 5:**
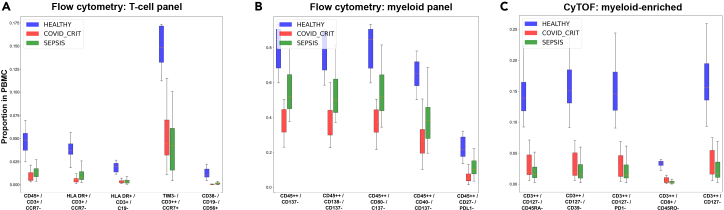
Immune cell signatures distinguishing COVID-19, sepsis, and healthy controls (A) Flow-cytometry analysis of T cell populations. (B) Flow-cytometry analysis of myeloid cell populations. (C) Mass-cytometry (CyTOF) analysis of myeloid-enriched cell populations. Boxplots show median and IQR; whiskers extend to 1.5× IQR. The five most significant marker combinations by one-way ANOVA are shown for each panel. Sample sizes: healthy controls, *n* = 10; COVID-critical, *n* = 18; sepsis, *n* = 22.

Our analysis (Figures 5A–5C) revealed distinct T cell populations with discriminatory power between COVID-19, sepsis, and healthy controls. Most notably, we identified a CD3^++^/CD45RA^++^/CD8^+^ population, likely representing naive cytotoxic T cells, and several CD4^+^ subsets with varying expression of activation markers (CD38 and CD25) and migration markers (CCR7). The CD4^+^/CD45RA^++^/CCR7^++^ signature suggests involvement of naive helper T cells, while CD4^+^ populations expressing high levels of CD38 and CD25 indicate increased T cell activation states. These findings highlight the differential immune responses in COVID-19 vs. sepsis, particularly in the naive and activation status of both CD4^+^ and CD8^+^ T cell populations (Figure 5).

## Discussion

We show here that batch alignment of cytometry data with an autoencoder neural network architecture achieves sufficiently normalized marker intensity signals to enable use of a multi-dimensional representation of this information in subsequent machine-learning classification tasks. This “hypervoxel” representation maintains interpretable marker-based features and establishes a new conceptual framework for working with large, integrated cytometry datasets for sample classification and predictive model-building tasks. Furthermore, the method eliminates the requirement for per-plate normal controls and manual gating, potentially increasing practical utility and analytical reproducibility. This provides a simplified path toward to automated analysis of very large cytometry datasets that are both longitudinal and multi-center.

A critical prerequisite for application of the hypervoxel methodology described here is the accurate alignment of samples to remove technical batch effects that are ubiquitous in cytometry data. The method presented herein incorporates batch alignment as an integral first step prior to a downstream classification task. In this way it differs from many current machine-learning approaches, including deep neural networks, which commonly struggle with prospective applications due to batch effects that are significantly outside the original model training distribution. By optimizing both reconstruction accuracy and distribution matching through a combined loss function, this normalization step aims to ensure that cells expressing similar marker levels will consistently fall within the same voxels, regardless of their batch of origin. The VoxelCoder approach integrates both batch alignment and the hypervoxel representation of cell counts and hence mitigates technical variations in future data acquisition, a key consideration in prospective clinical applications.

We benchmarked our autoencoder-based batch-alignment approach using a controlled murine dataset with synthetically introduced batch effects, demonstrating that VoxelCoder effectively addresses technical and batch-related variation in cytometry data. Critically, VoxelCoder retains more biological signal than popular methods, including CytoNorm^9^^,^^10^ and Harmony,^13^ as evidenced by superior AUC values and statistical significance of identified features in real experimental datasets. This performance highlights the critical dual requirement in batch correction: minimizing technical variation while simultaneously preserving biological signal. The autoencoder framework’s ability to handle non-linear batch effects while maintaining population structure preserves the resolution of input data, enabling robust analysis of large-scale studies. This strength is best demonstrated in downstream applications, including two challenging clinical datasets: predicting CMV serostatus from cytometry data spanning nine independent studies and discriminating between COVID-19 and sepsis patients in the COMBAT study across six experimental batches.

Our approach achieves classification performance comparable to that of black-box machine-learning methods on input flow cytometry and CyTOF datasets that were not considered valuable for classification tasks by the original projects that generated them. Unlike black-box machine-learning classifiers, our multi-dimensional representation of normalized data maintains complete interpretability of cellular counts and marker intensity features. This represents an important advantage over deep-learning approaches that require complex post hoc analysis. The hypervoxel analysis framework complements rather than replaces manual gating, systematically exploring the multi-dimensional marker space to identify significant populations for validation through conventional gating strategies while enabling comprehensive examination of marker combinations that might be overlooked in manual analysis. Finally, unlike existing methods that require common controls across batches, our method achieves normalization by learning marker distributions from a reference batch, making it applicable to studies where matched samples are unavailable. This capability is particularly valuable for integration of public datasets, where matched controls are often not available across different studies.

For this work, we generated a benchmarking dataset for the evaluation of the performance of batch-alignment methodology. We have applied this here to benchmark the performance of our tool, VoxelCoder, with three other prominent batch-normalization tools: CytoNorm,^9^^,^^10^ CyCombine,^12^ and Harmony.^13^ As the cells in each technical replicate for each mouse were derived from the same original splenocyte suspension, a perfect batch alignment would result in little or no difference between technical replicates. We find that VoxelCoder achieves alignment performance equivalent to that of CyCombine and Harmony for this task. Furthermore, a gender bias between the biological replicate mice appeared to skew some batch-alignment results. Taking advantage of subtle cellular phenotypes, such as differences identified between male and female mice,^28^ we observed that VoxelCoder removed less true biological signal during batch alignment than other tools, especially Harmony. This represents an important facet of cytometry batch normalization, especially when applying this to detection of subtle cellular phenotypes of human diseases.

When applied to a CMV serostatus prediction task, the VoxelCoder method identified populations consistent with previous findings^25^^,^^29^ while providing refined phenotypic resolution. Specifically, the hyperdimensional representation identified CD8^+^/CD3^+^/CD27^−^ T cells as the most highly predictive population (*p* < 10^−20^), with the top five predictive marker combinations all featuring CD8^+^ cells with low CD27 expression, four of which also showed low CCR7 expression. This aligns with the CD3^+^/CD8^+^/CD27^−^/CD94^+^ population reported by Hu et al.,^24^ confirming CD27 downregulation as a core component of the CMV-associated signature. However, our analysis suggests that CD27 and CCR7 downregulation alone provide robust predictive power. Similarly, in the COVID-19/sepsis classification task, the method revealed distinctive T cell activation signatures that differentiated between disease states while maintaining direct interpretability. The original study did not identify these signatures, as shown by their reported discriminative features.

Future work could explore two new directions. First, the development of adaptive thresholding approaches for marker discretization could improve robustness across different experimental contexts. Second, systematic validation studies comparing our voxel-based populations against published manual gating strategies using open-access datasets could help quantify the correspondence between these approaches. Specifically, analyzing how frequently cells within established manual gates map to specific hypervoxels could provide valuable insights into the relationship between these different analysis paradigms.

We show that batch alignment with autoencoders allows a simple representation that improves classifier performance. Through effective batch alignment, larger bodies of cytometry data may be now integrated and interpreted while preserving the underlying biological signal structure. This enables data science approaches for longitudinal studies, multi-center cohorts, and large-scale cytometry studies conducted across extended timescales or with heterogeneous equipment. This will also allow better integration of datasets from public repositories of flow-cytometry and CyTOF mass-cytometry datasets.

## Methods

### Generation of benchmark mouse synthetic batch-effect flow-cytometry dataset

A benchmarking dataset was produced from flow cytometry of three biological replicate wild-type C57BL/6 mice (two female, one male), two mutant C57BL/6 strains (*Kenobi*, a CD79a null strain^30^), and a RAG-1 null strain.^31^ The dataset was generated from splenic cell suspensions from each mouse split to three technical replicates and prepared such as to introduce a controlled synthetic batch effect from the same input cells. The synthetic batch effect was produced through minor manipulation of antibody dilutions in three marker panels (see below and Table 4).

**Table 4:** Details of antibodies and dilutions used for each panel in the mouse synthetic batch-effect dataset

Marker	Fluorophore	Panel 1 dilution factor	Panel 2 dilution factor	Panel 3 dilution factor	Supplier	Catalog #
CD3	FITC	200	200	300	BioLegend	100204
CD25	PE	400	400	400	BioLegend	102008
CD8	CD8	100	400	400	BD	563786
CD44	PacBlue	100	400	400	BioLegend	103020
CD62L	BV605	400	600	600	BioLegend	104438
CD19	BV510	600	800	800	BioLegend	115546
IgD	PerCPCy5.5	200	800	800	BD	564273
IgM	R718	100	100	600	BD	752171
NK1.1	APC	200	400	600	BD	550627
Ly6C	PECy7	200	200	400	BioLegend	128018
B220	BUV737	300	600	800	BD	612838
CD4	AF700	200	300	600	BioLegend	100430
Live/Dead	ef780	400	600	800	Thermo Fisher	65-0865-14

Each antibody is described by the marker to which it binds, the attached fluorophore, the dilution factor in each panel, and the catalog number and supplier of the antibody. Panel 1 is the optimal antibody concentrations determined by antibody titration, with minor changes in the further two panels to induce a synthetic batch effect.

Mouse spleens were collected into 3 mL of fluorescence-activated cell sorting (FACS) buffer (2.5% fetal bovine serum, 0.1% sodium azide, 0.01% EDTA, and 10% PBS) and mashed through a 70-μm cell strainer. Cells were transferred into 15-mL Falcon tubes and centrifuged at 465 × *g* for 5 min at 4°C, following which the supernatant was discarded. Red blood cells were lysed by resuspending the pellet in 3 mL of 1× lysis buffer (Thermo Fisher Scientific, 00-4300-54) and incubated for 1 min at room temperature. After incubation, lysis buffer was diluted by the addition of 7 mL of FACS buffer and centrifuged at 465 × *g* for 5 min at 4°C. The supernatant was subsequently discarded and the cells washed by resuspending in 10 mL of FACS buffer before being centrifuged again at 465 × *g* for 5 min at 4°C. The cell pellet was resuspended in 0.5 mL of FACS buffer and the total cell count calculated using the Luna-II Automated Cell Counter (Thermo Fisher Scientific). Cells were plated into a 96-well round-bottom plate.

Cells were blocked in 25 μL of 2× Fc block (BD, Ms CD16/CD32 Pure 2.4G2, 553142) for 5 min before the addition of 25 μL of 2× Live/Dead stain (Thermo Fisher Scientific, fixable viability dye E780, 65-0865-14). After staining, cells were washed in 200 μL of 1× PBS and centrifuged at 465 × *g* for 5 min at 4°C. For each biological replicate mouse, the total cell suspensions were divided into three equal parts. Cells were stained with 50 μL of antibody cocktail (in brilliant stain buffer [BD Biosciences, 566349]) with panels diluted to different antibody concentrations (Table 4) or the respective single-color control at 4°C for 30 min before washing in FACS buffer. Cells were fixed using Fix buffer (eBioscience, 00-5523-00) according to the manufacturer’s instructions. After fixing, cells were washed twice with FACS buffer before resuspending in 80 μL of FACS buffer for acquisition on the LSRFortessa X-20 (BD).

### Dataset pre-processing

Flow-cytometry data were processed using a custom Python function built on the FlowKit framework.^32^ The function accepts Flow Cytometry Standard (∗.fcs) files and performs sequential pre-processing steps. First, compensation was applied to correct for fluorescence spillover between channels. Cellular debris and non-single-cell events were then removed using sequential polygonal gates defined in the forward and side-scatter dimensions. For flow-cytometry data, a logicle (biexponential) transformation with parameters *T* = 262,144, *W* = 0.5, *M* = 4.5, and *A* = 0 was applied, while mass-cytometry (CyTOF) data were transformed using arcsinh transformation with a cofactor of 5 followed by scaling by a factor of 1/8. These transformations, which are standard practice in cytometry analysis, provide appropriate scaling for both negative and positive values while maintaining resolution of low-intensity signals. For training the autoencoder batch-alignment model, a fixed count of 30,000 cells were subsampled from each sample in the reference batch.

The synthetic batch dataset is available via Zenodo.^33^ CMV data were sourced from the repository of Hu,^34^ and COMBAT COVID-19 data were sourced from the public repository made available by the study authors.^27^

### Batch alignment via deep neural network autoencoder

The batch-alignment method was implemented as a custom PyTorch^35^ model using an autoencoder neural network architecture. The network consisted of three encoding layers (64, 32, and 16 nodes) and three symmetrical decoding layers, with ReLU activation functions between layers. The autoencoder was trained to transform input data from different batches to match a reference distribution while preserving biological signal. One batch was randomly selected as the reference, and the model was trained using a combination of MSE loss and a custom histogram loss function that encouraged the transformed data to maintain marker distribution shapes similar to those of the reference batch.

The histogram loss term was computed by comparing normalized histogram distributions between the network output and reference data across all markers, using Wasserstein distance as the similarity metric. This dual loss function helped ensure that both individual cell measurements and overall population distributions were preserved during batch alignment. The model was trained for 1,200 epochs using the Adam optimizer^36^ with a learning rate of 0.002 and a batch size of 1,024 cells. The histogram loss term was weighted by a factor *β* = 0.002, which was gradually decreased during training to allow fine-tuning of individual cell measurements in later epochs while maintaining population-level distribution matching.

### Voxel gating for multi-marker population analysis

Following batch alignment, we implemented a systematic voxel-based analysis strategy to characterize cellular populations. For each marker, expression values were discretized into three levels (LOW, MED, and HIGH) using fixed-intensity thresholds (<0.3, 0.3–0.6, and >0.6 in normalized intensity units). We then systematically generated all possible three-marker combinations from the panel. This exhaustive strategy enables unbiased discovery of cellular phenotypes without prior assumptions regarding biological hierarchy. For each marker combination, we computed the proportion of cells falling within each possible discretized state combination, creating a high-dimensional feature vector of population frequencies. Each combination of three markers generated 27 possible states (3^3^ combinations of LOW/MED/HIGH), with the frequency of cells in each state serving as a feature for downstream analysis.

These population frequency features were compiled into a sample-by-feature matrix where each row represented a sample and each column represented the frequency of cells in a particular marker combination state. This matrix served as input for subsequent machine-learning classification tasks, providing a comprehensive yet interpretable representation of the cellular composition of each sample. This approach effectively transforms complex single-cell data into discrete population-based features while maintaining the ability to examine high-dimensional marker relationships. Voxel features with maximum occupancy below 1% across all samples were excluded to filter out sparse features representing potential staining artifacts or statistical noise.

### Classification and identification of significant features

Following voxel-based feature generation, we implemented machine-learning classification approaches tailored to each dataset. For the CMV dataset,^25^^,^^29^ we employed a hold-out validation strategy, designating a specific batch (SDY519) as the test set while training on all remaining batches. For the COVID-19 dataset,^26^^,^^27^ we implemented batch-wise cross-validation, where the model was trained on *n* − 1 batches and tested on the remaining batch. In both cases, we used XGBoost classifiers^37^ to distinguish between patient groups (CMV-positive vs. CMV-negative for the first dataset; and healthy controls, COVID-19 critical patients, and sepsis patients for the second dataset) using population frequencies derived from our voxel-gating analysis. Features were standardized using *Z*-score normalization, and class weights were applied to address class imbalance. The models were optimized using 500 trees with a maximum depth of 6, and thresholds for prediction were fine-tuned using F1 scores.

Significant features were identified through a combination of statistical testing and machine-learning importance metrics. Initial feature selection was performed using Mann-Whitney U tests (two-group comparisons) or one-way ANOVA (multi-group comparisons) to identify marker combinations with significant differences between groups.

## Resource availability

### Lead contact

Requests for further information and resources should be directed to and will be fulfilled by the lead contact, Dr. Benjamin Mashford (benjamin.mashford@anu.edu.au).

### Materials availability

This study did not generate new unique reagents.

### Data and code availability

The VoxelCoder code is available at GitHub (https://github.com/ben-mashford/voxelcoder_CLI) and archived on Zenodo.^24^ The synthetic batch-effect mouse cytometry dataset generated for this study is available on Zenodo.^33^ The CMV dataset was obtained from Hu.^34^ and is available at GitHub (https://github.com/hzc363/DeepLearningCyTOF). The COMBAT COVID-19 dataset is publicly available from the original study authors.^27^

## Acknowledgments

The authors thank the National Computational Infrastructure (Australia) for continued access to significant computation resources, and the Cytometry, Histology, and Advanced Spatial Multiomics Facility at the John Curtin School of Medical Research. T.H. acknowledges the support of 10.13039/501100023560Bioplatforms Australia. This work has been partly funded by the 10.13039/501100025520Medical Research Future Fund (Australia) through grants MRF2016149 (M.M. and T.D.A.) and ARG76376 (A.R.D., F.-J.L., K.K., and K.E.M.D.) T.D.A. and B.S.M. have been supported by the Jubilee Joint Fellowship scheme of the 10.13039/501100000995Australian National University. We thank the National Computational Infrastructure (Australia) for continued access to significant computation resources and technical expertise.

## Author contributions

B.S.M., D.H., and T.D.A. designed research; B.S.M., T.H., M.M., A.J., Z.Z., and T.D.A. performed research; A.R.D., K.E.M.D., F.-J.L., K.K., S.H.R., and D.H. generated data; and B.S.M and T.D.A. wrote the paper.

## Declaration of interests

The authors declare no competing interests.

## Supplemental information

Document S1. Figures S1–S4 and Tables S1 and S2

Document S2. Article plus supplemental information
