# Supplementary material for: VoxelCoder: Classification of human cellular phenotypes via autoencoder batch alignment and hyperdimensional representation of cytometry data
Source: Patterns (N Y). 2026 Mar 26;7(5):101511. doi: 10.1016/j.patter.2026.101511 (PMC13161691; doi:10.1016/j.patter.2026.101511)
Supplement: Document S1. Figures S1–S4 and Tables S1 and S2 [file mmc1.pdf]

Patterns, Volume *7*

Supplemental information

VoxelCoder: Classification of human cellular phenotypes via autoencoder batch alignment and hyperdimensional representation of cytometry data

Benjamin S. Mashford, Timothy Hewitt, Maryam May, Zixin Zhuang, Akshat Jain, Koula E.M. Diamand, Fei-Ju Li, Kristy Kwong, Stuart H. Read, Ainsley R. Davies, Dillon Hammill, and T. Daniel Andrews

SUPPLEMENTARY INFORMATION

Supplementary Figure 1. Scatter plots showing the performance of four different batch normalization methods (VoxelCoder, CyCombine, CytoNorm, and Harmony) alongside non-batch-aligned controls for Mouse #1, Batch B. The top row shows a scatter plot of CD8 vs. CD4, while the lower row shows CM19 vs. CD3.

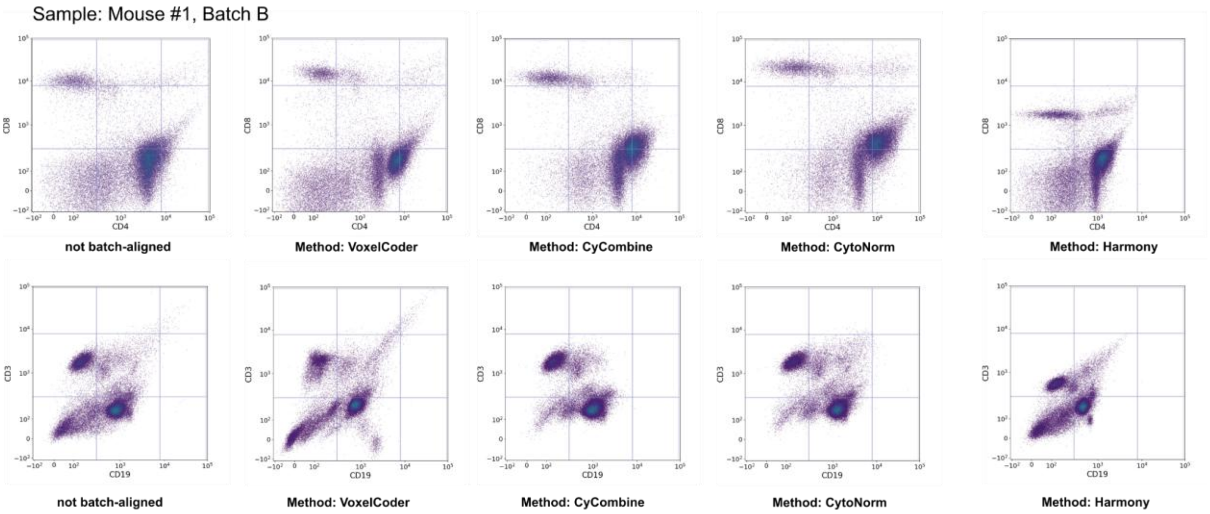

Supplementary Figure 2: Flow cytometry analysis and machine learning classification of immune cell populations across patient groups in COMBAT dataset (T-cell panel). **(a)** Histogram distributions of key T-cell surface markers (CD3, CD4, CD8) and activation/migration markers (CD45, CCR7, CD25), showing expression patterns with negative (pink), intermediate (green), and positive (purple) gating intervals. **(b)** Scatter plots demonstrating the co-expression relationships between paired markers, including T-cell defining markers (CD3 vs CD4, CD3 vs CD8) and activation markers (CD45 vs CCR7, CD69 vs CD25). **(c)** Receiver Operating Characteristic (ROC) curves showing the classification performance for distinguishing between healthy controls, COVID-19 critical patients, and sepsis patients, with area under the curve (AUC) values indicated for each group. **(d)** Confusion matrix displaying the classification accuracy of the model across the three patient groups, with percentages indicating correct and incorrect classifications.

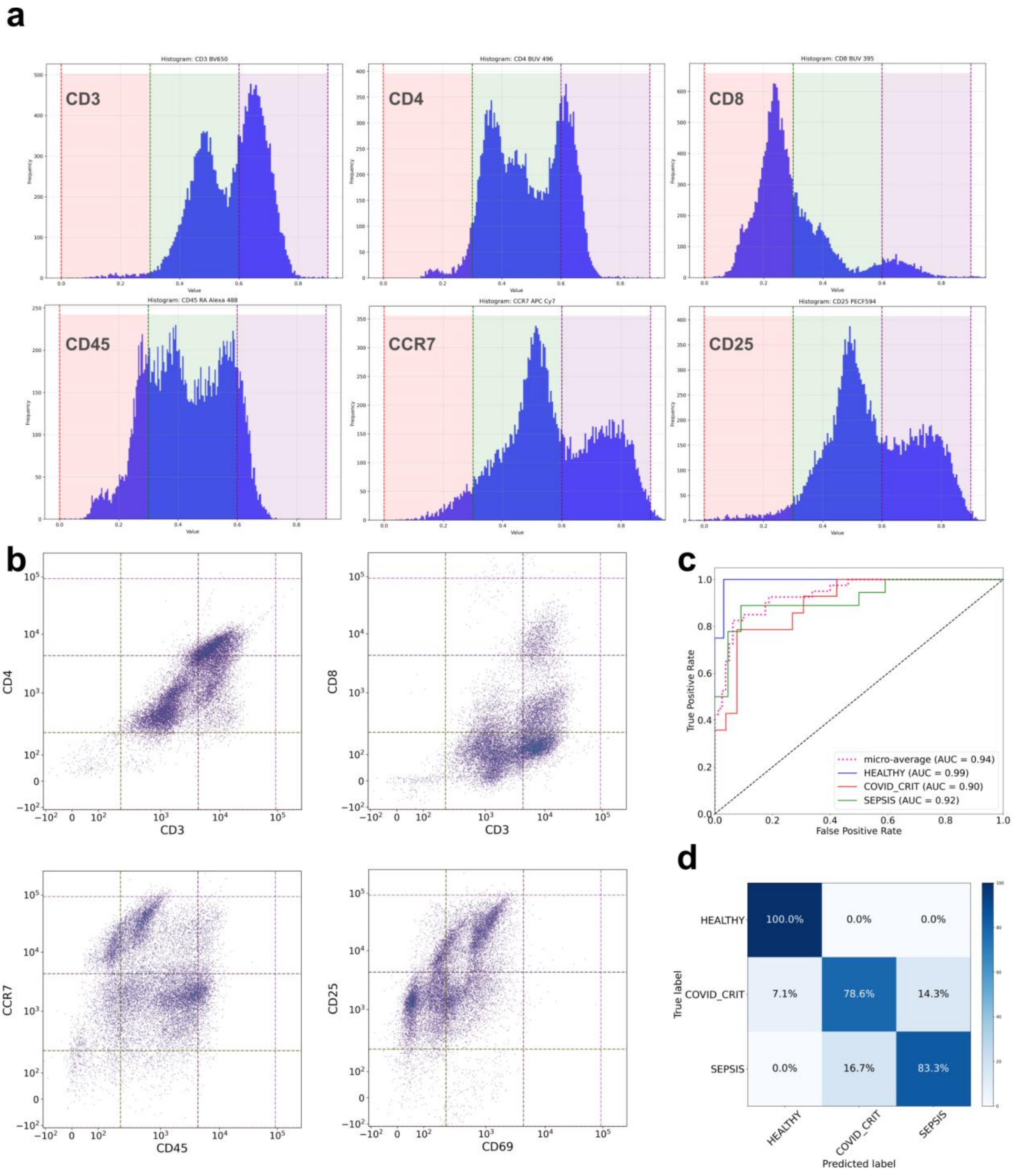

Supplementary Figure 3. XGBoost classifier performance on CMV-status discrimination task, comparing a). VoxelCoder-aligned dataset vs. b). Harmony-aligned dataset.

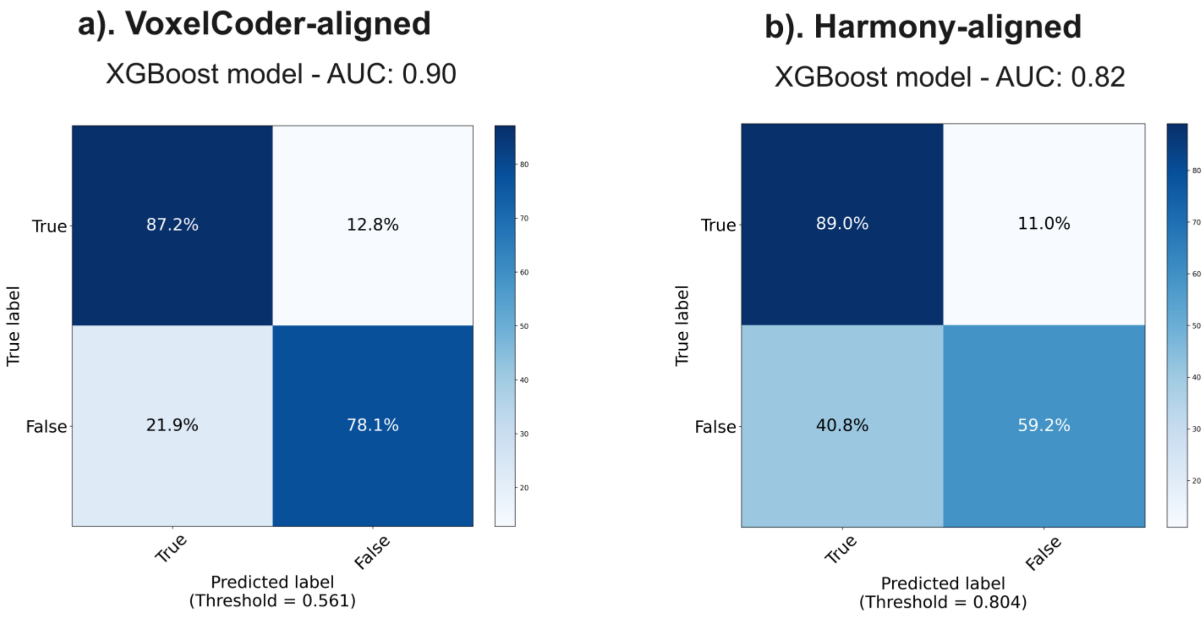

Supplementary Figure 4. Sensitivity analysis of reference batch selection on downstream classification performance. Area under the ROC curve (AUC) values for CMV serostatus prediction when using four different batches as the reference batch for autoencoder-based batch alignment.

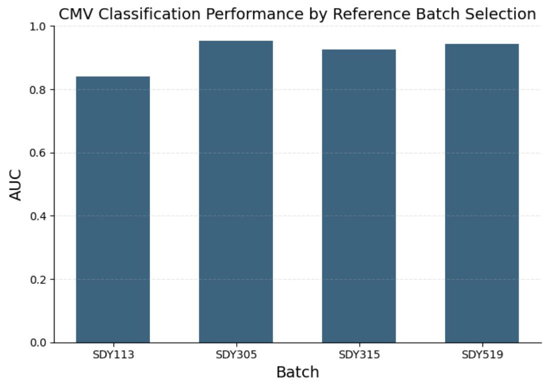

Supplementary Tables

Classification	Sample count
COVID (severe)	40
Sepsis	22
COVID (mild)	18
COVID (critical)	18
COVID (health care worker)	13
Flu	11
Healthy volunteer	10
LDN treated	2
**Total**	**134**

Supplementary Table 1. Distribution of patient samples in entire COMBAT dataset across disease categories and control groups.

Statistical Metric	Harmony-aligned	VoxelCoder-aligned
Mean Diagonal Kernel MMD	0.011	0.016
Mean Diagonal Energy Distance	2×10^{-4}	6×10^{-4}
MANOVA on PCs (*F*-statistic)	0.01 ($p = 0.94$)	0.04 ($p = 0.84$)
Spearman Correlation	0.624	0.770
Male Rag Strain KL Divergence	0.472	0.337

Supplementary Table 2. Comparison of distribution matching and structure preservation statistics between VoxelCoder and Harmony using the synthetic batch-effect dataset.
